# Supplementary material for: Development of a Porcine Cell Line Stably Expressing Ephrin‐B2 for Nipah Virus Research and Diagnostic Testing
Source: Microbiol Immunol. 2025 Nov 17;70(1):36–46. doi: 10.1111/1348-0421.70022 (PMC12773662; doi:10.1111/1348-0421.70022)
Supplement: Supplementary file 1 — Supporting Text 1: Synthesized DNA for generating a plasmid expressing pig ephrin‐B2. The coding sequence of pig ephrin‐B2 was synthesized according to the amino acid sequence deposited in GenBank (accession number: ABV44489.1) with codon optimization to pig cells. The start and stop codons are underlined. Supporting Text 2: Synthesized DNA for generating a plasmid expressing Nipah virus (NiV) G and F. The coding sequences of NiV G and F proteins were synthesized according to the amino acid sequences deposited in GenBank (accession numbers: AAK29088.1 and AAK29087.1). The start and stop codons are underlined. [file MIM-70-36-s002.pdf]

S1 Text 1. Synthesized DNA for generating a plasmid expressing pig Ephrin-B2

<u>ATG</u>GCAGCTAGAAGGGACAGCGTCTGGAAGTATTGTTGGGGTGTTTTGATGGTGCTCTGT
CGCACAGCAATTTCAAGAAGTATAGTTCTTGAACCGATCTATTGGAATAGCTCTAACTCAA
AGTTCCTTCCTGGACAGGGACTGGTCCTTTACCCACAGATCGGGGATAAGCTGGATATAA
TTTGCCCGAAGGTCGACAGTAAGACCGTGGGACAATATGAGTATTACAAAGTGTATATGG
TGGATAAGGATCAGGCCGATCGCTGCACGATTAAGAAAGAGAACACGCCTCTCCTGAATT
GTGCTAGACCTGACCAGGATGTCAAATTCACAATCAAGTTTCAAGAATTTAGCCCGAACC
TGTGGGGGCTCGAATTTCAGAAGAACAGGGATTACTATATCATTTCAACGAGCAACGGTT
CCCTCGAGGGATTGGACAATCAAGAAGGGGGCGTCTGCCAAACACGCGCGATGAAAATA
CTGATGAAAGTGGGACAAGACGCCAGCAGTGCCGGTTCTACCAGACATAACGAACCGAC
AAGACGGCCTGAACTCGAGGCGGGCACGAACGGTCGCTCTAGTACGACGAGTCCTTTTG
TCAAGCCAAATCCTGGGTCCAGTACAGATGGGAACAGCGCGGGCCATAGTGGTAATAATA
TTCTGGGTTCCGAAGTTGCGCTCTTTGCCGGCATCGCGTCAGGCTGCATCATTTTCATTGT
GATTATTATCACACTCGTTGTCCTCTTGCTGAAATATAGGAGGAGGCACCGGAAGCATTCC
CCCCAACACACTGCCACCCTCTCTATCTCAACGTTGGCTACTCCGAAGAGGGGTGGGAAT
AACAATGGCAGCGAGCCTTCCGACATTATTATTCCATTGAGAACGGCAGATAGTGTGTTCT
GCCCGCACTATGAGAAGGTCAGCGGTGACTATGGACATCCAGTTTACATAGTGCAAGAAA
TGCCCCCGCAGTCCCCTGCCAATATTTACTATAAAGTCGAGCAGAAGCTTATTTCCGAAGA
AGATCTC<u>TAA</u>

S1 Text 2. Synthesized DNA for generating a plasmid expressing Nipah virus G and F proteins.

Nipah virus G protein

ATGCCAGCGGAGAATAAGAAAGTGAGATTCGAGAATACTACGAGCGACAAAGGCAAGAT
ACCCAGCAAGGTAATAAAAAGCTACTATGGTACAATGGACATAAAAAAAATAAACGAAG
GGCTCCTGGACAGCAAGATCCTTTCAGCTTTTAACACGGTGATAGCACTCTTGGGCTCAA
TAGTGATTATCGTGATGAACATAATGATTATTCAGAATTACACTCGGAGTACTGATAATCAA
GCAGTCATAAAGGATGCTCTTCAGGGCATCCAACAACAGATAAAAGGCCTTGCCGACAA
AATCGGTACAGAAATTGGACCAAAAGTCTCACTTATAGACACCTCCTCAACAATAACCAT
CCCTGCTAATATTGGGCTCCTCGGCAGTAAGATCTCTCAGTCAACAGCGAGCATCAATGA
AAATGTGAACGAAAAGTGCAAGTTCACTCTGCCACCCCTGAAGATTCATGAGTGTAACAT
AAGCTGTCCGAATCCACTCCCTTTTCGGGAGTATAGGCCGCAAACTGAGGGAGTGAGCA
ACCTGGTGGGATTGCCGAACAACATCTGCTTGCAAAAGACGAGTAATCAGATTCTGAAG
CCGAAACTCATATCCTACACACTGCCAGTTGTCGGACAGAGTGGTACGTGTATCACCGAT
CCTCTTCTGGCGATGGATGAAGGGTATTTTGCATATTCCCATCTGGAACGCATCGGCTCCT
GCAGTCGAGGGGTATCCAAGCAGCGAATCATTGGTGTCGGCGAAGTGTTGGATCGAGGA
GATGAAGTGCCAAGTTTGTTCATGACGAACGTGTGGACCCCGCCAAATCCCAACACCGT
TTACCACTGTTCAGCTGTTTACAATAACGAGTTCTACTACGTTCTCTGTGCAGTGTCAACC
GTGGGCGACCCAATCCTTAATTCTACTTACTGGAGCGGGTCCCTTATGATGACAAGGCTC
GCCGTGAAACCGAAGAGTAACGGAGGGGGTTATAATCAACATCAGCTCGCCCTTCGCTCT
ATAGAAAAGGGCAGATACGATAAGGTTATGCCTTACGGTCCTAGCGGTATCAAGCAAGGA
GATACCCTTTATTTCCCCGCTGTAGGTTTTCTCGTCCGCACGGAGTTCAAATATAATGATTC
CAACTGTCCAATTACTAAGTGCCAATACTCCAAACCCGAGAATTGTAGATTGAGTATGGG
GATACGGCCCAACAGCCATTACATCCTGAGATCTGGGTTGCTGAAATACAACCTGTCAGA
TGGCGAGAACCCTAAAGTGGTCTTTATTGAGATCTCAGATCAGCGGTTGTCAATCGGGAG
TCCCAGCAAGATCTATGATTCTCTCGGACAGCCGGTCTTCTACCAGGCAAGCTTTTCATGG
GACACCATGATCAAATTTGGTGACGTTCTTACGGTAAATCCGCTTGTGGTAAACTGGCGC
AATAATACTGTGATCAGTCGGCCAGGTCAATCTCAATGCCCGCGCTTCAATACATGCCCCG
AGATCTGTTGGGAAGGTGTATACAACGACGCATTCCTGATCGACAGGATTAACTGGATAA
GCGCCGGAGTTTTCCTTGACTCAAACCAAACTGCTGAAAACCCAGTTTTCACAGTTTTCA
AAGACAACGAAATCTTGTATCGGGCACAGTTGGCCTCAGAGGATACGAATGCTCAAAAA
ACAATCACAAACTGTTTCCTCCTTAAGAACAAGATCTGGTGCATCAGCTTGGTAGAGATA
TACGACACCGGTGACAACGTTATCCGGCCAAAACTTTTCGCCGTAAAAATACCCGAACAA
TGCACGTATCCCTATGATGTCCCCGACTATGCTTAG

Nipah virus F protein

ATGGTAGTGATTCTTGACAAAAGGTGTTACTGCAATCTTCTTATTCTTATACTTATGATCTC

CGAATGCTCTGTGGGAATCCTGCACTACGAAAAGCTCAGTAAGATTGGGCTCGTCAAGG
GAGTTACCAGGAAGTACAAGATCAAATCTAATCCACTCACAAAGGACATAGTTATTAAGA
TGATTCCCAACGTCTCCAATATGTCACAGTGCACAGGGAGTGTAATGGAAAATTACAAAA
CAAGACTCAATGGCATCTTGACGCCGATAAAGGGTGCGCTTGAGATATATAAAAATAACA
CTCACGACTTGGACTACAAGGATGATGACGATAAGGTTGGAGACGTGAGATTGGCCGGA
GTTATTATGGCCGGTGTCGCTATTGGTATCGCGACCGCGGCCCAGATTACGGCAGGAGTAG
CGCTGTACGAGGCCATGAAGAATGCGGACAATATCAACAAACTCAAGAGTTCCATAGAA
AGTACTAATGAGGCTGTTGTGAAACTGCAAGAGACAGCTGAGAAGACTGTCTATGTATTG
ACCGCGTTGCAAGACTATATTAACACTAACCTCGTTCCGACAATAGACAAGATAAGTTGTA
AGCAAACTGAGCTTAGCTTGGACCTGGCATTGTCCAAGTACCTTAGCGATCTGCTGTTTG
TATTTGGTCCGAACTTGCAGGACCCAGTATCTAACTCAATGACTATTCAGGCTATATCCCA
GGCTTTCGGCGGGAACTACGAAACACTTTTGAGGACGCTCGGGTACGCAACTGAGGACT
TTGATGATCTCCTGGAGTCTGATTCTATTACGGGACAGATAATCTACGTTGATCTCAGTTCT
TATTATATAATCGTGCGCGTCTATTTCCCGATTCTCACTGAAATTCAACAAGCGTACATACA
GGAGCTGCTGCCCGTGAGCTTCAACAATGATAACAGCGAATGGATCAGTATCGTGCCAAA
CTTCATCTTGGTCAGAAACACCTTGATTAGTAACATAGAGATCGGATTTTGTCTGATCACT
AAGCGCAGTGTCATCTGCAATCAAGATTACGCAACTCCAATGACCAACAATATGAGAGAA
TGTTTGACTGGGTCCACAGAAAAATGTCCAAGAGAGCTTGTGGTGTCTTCCCACGTGCC
GCGGTTTGCCTTGAGCAACGGAGTACTTTTCGCGAATTGTATTTCAGTCACCTGCCAATGT
CAGACAACGGGTCGAGCAATATCACAGTCAGGGGAGCAAACCCTGCTGATGATAGATAAT
ACCACCTGTCCTACTGCCGTGCTCGGCAATGTCATCATTTCTTTGGGCAAATACCTTGGAT
CAGTTAATTACAATTCAGAGGGTATTGCAATAGGCCCTCCGGTCTTCACGGATAAGGTTGA
TATTTCTTCACAGATTAGTAGTATGAATCAGTCTCTGCAGCAGTCTAAGGACTATATAAAA
GAGGCACAACGCCTCCTTGACACCGTGAACCCTTCACTGATAAGCATGCTTAGCATGATA
ATTCTGTATGTTTTGAGTATCGCAAGTCTTTGTATAGGACTTATTACGTTTATAAGTTTTATT
ATAGTTGAGAAAAAAAGAAATACCTACTCTAGATTGGAGGACAGGAGAGTGAGACCTAC
TTCATCCGGAGATCTCTACTACATTGGCACCGACACCTACAGGTATATTTGA
