# Supplementary material for: Development of a Porcine Cell Line Stably Expressing Ephrin‐B2 for Nipah Virus Research and Diagnostic Testing
Source: Microbiol Immunol. 2025 Nov 17;70(1):36–46. doi: 10.1111/1348-0421.70022 (PMC12773662; doi:10.1111/1348-0421.70022)

Supplementary Figure 1

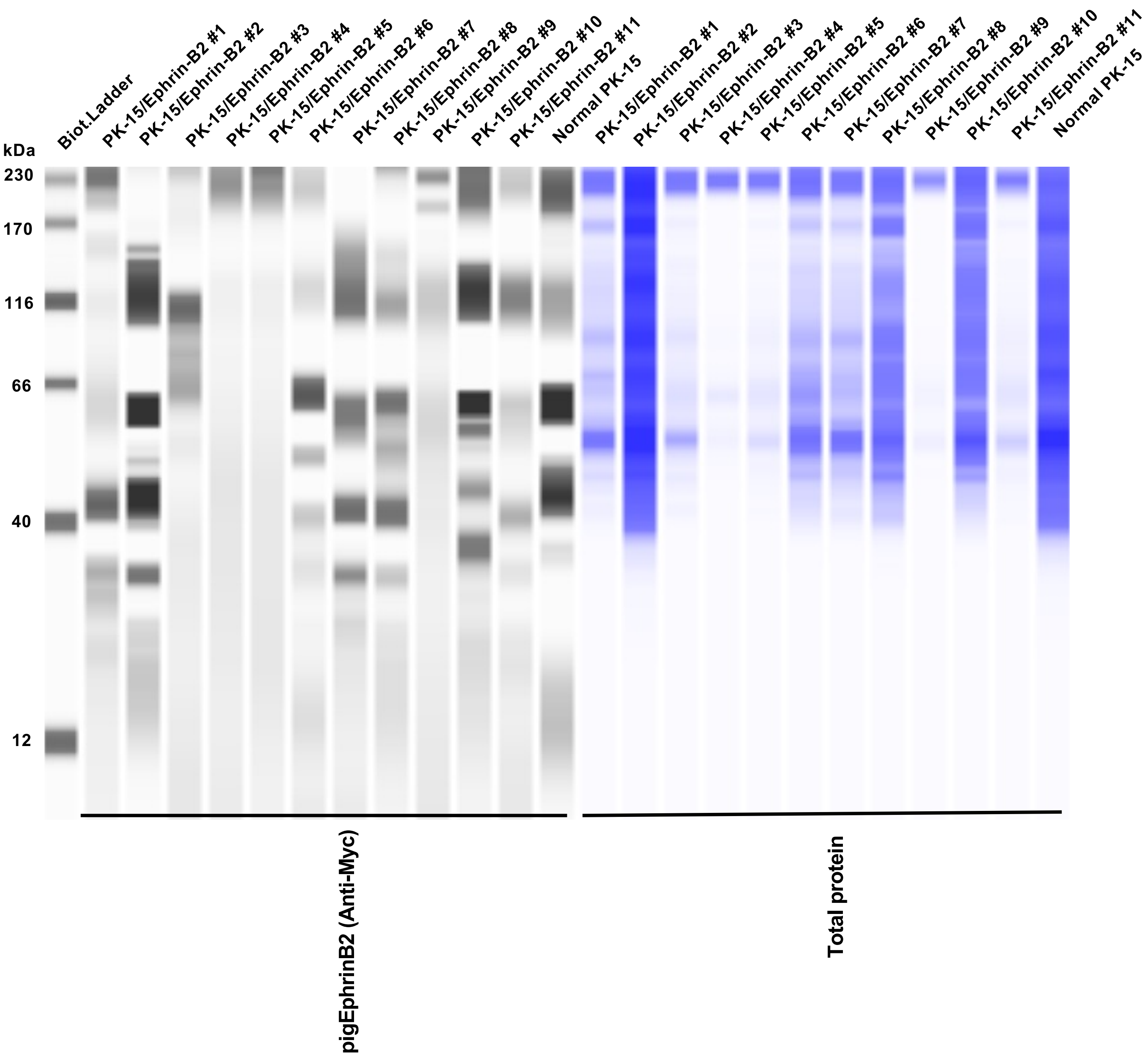

Sample	Peak1	Relative expression (fold) (Sample / Normal PK-15)
Noraml PK-15	32.1	1
PK-15/Ephrin-B2 #1	87.4	2.7
PK-15/Ephrin-B2 #2	0	0.0
PK-15/Ephrin-B2 #3	733.9	22.9
PK-15/Ephrin-B2 #4	367.3	11.4
PK-15/Ephrin-B2 #5	36.9	1.1
PK-15/Ephrin-B2 #6	1365.5	42.5
PK-15/Ephrin-B2 #7	58.9	1.8
PK-15/Ephrin-B2 #8	0.9	0.0
PK-15/Ephrin-B2 #9	21.3	0.7
PK-15/Ephrin-B2 #10	203.2	6.3
PK-15/Ephrin-B2 #11	73.8	2.3

Supplementary Figure 2

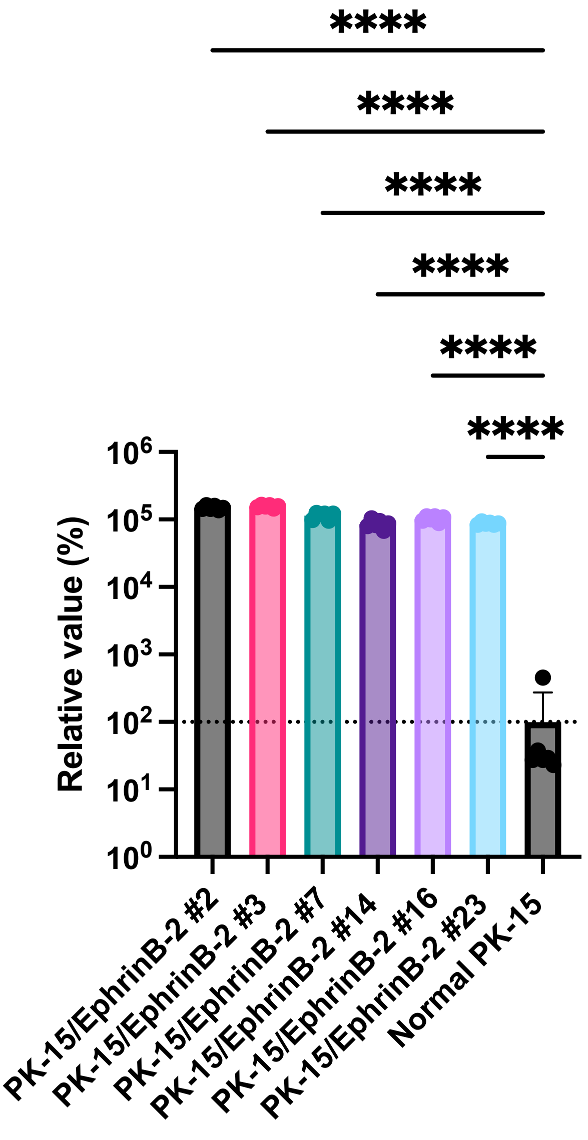

Supplement: Supplementary file 2 — Supporting Figure 1: Expression screening of PK‐15/Ephrin‐B2 cell clones. Expression of cellular myelocytomatosis oncogene product (Myc)‐tagged pig ephrin‐B2 in PK‐15 cells was evaluated by western blotting. The cellular lysate of normal PK‐15 cells was used as a negative control (empty). Cellular lysates were probed with an anti‐Myc antibody (left) and then re‐probed with the Total Protein Detection Module (right) as a loading control. The calculated expression level for each clone is presented below the figure. Supporting Figure 2: Functional screening of PK‐15/Ephrin‐B2 cell clones. Normal PK‐15 cells or PK‐15/Ephrin‐B2 cell clones were infected with the NiV pseudovirus. The luminescent signal was measured 2 days after infection. The relative value was calculated according to that in normal PK‐15 cells. Differences between normal PK‐15 cells and PK‐15/Ephrin‐B2 cell clones infected with the NiV pseudovirus were examined using a two‐tailed, unpaired Student's t‐test. ****p < 0.0001. [file MIM-70-36-s001.pdf]
